# Reproductive patterns among twins - a Swedish register study of men and women born 1973-1983

**DOI:** 10.1186/1471-2393-13-6

**Published:** 2013-01-16

**Authors:** Marie Bladh, Ann Josefsson, John Carstensen, Orvar Finnström, Gunilla Sydsjö

**Affiliations:** 1 Division of Obstetrics and Gynaecology, Department of Clinical and Experimental Medicine, Faculty of Health Sciences, Linköping University, Department of Obstetrics and Gynaecology in Linköping, County Council of Östergötland, Linköping, Sweden; 2 Division of Health and Society, Department of Medical and Health Sciences, Faculty of Arts and Science, Linköping University, Linköping, Sweden; 3 Division of Paediatrics, Department of Clinical and Experimental Medicine, Faculty of Health Sciences, Linköping University, Department of Paediatrics in Linköping, County Council of Östergötland, Linköping, Sweden; 4 Department of Obstetrics and Gynaecology, University Hospital, Linköping, SE 581 85, Sweden

**Keywords:** Twin, Singleton, Reproduction rate, Birth characteristics

## Abstract

**Background:**

During the last decades there has been a steady increase of twin births. A combination of improved medical treatment of preterm and small-for-gestational age children has contributed to a higher number of surviving twins. Prematurity is known to affect reproduction in a negative way. Few studies have focused on the potential effect twinning may have on future reproduction. Thus, the aim of this study was to investigate the effect of being born a twin compared to being born a singleton have on future reproduction.

**Methods:**

In a national population-based register study, all individuals born between 1973–1983 who were alive and living in Sweden at 13 years of age (n = 1 016 908) constituted the sample. Data on each study subject’s own birth as well as the birth of their first offspring, and parental socio-demographic factors were collected from Swedish population based registers. Hazard ratios and corresponding 95% CI was calculated using Cox proportional hazards model.

**Results:**

Twins, both men and women, had a reduced likelihood of reproducing compared to singletons (women: HR = 0.89, 95% CI = 0.86-0.93; men: HR = 0.92, 95% CI = 0.87-0.97). This difference in birth rates can only partly be explained by diverging birth characteristics. Amongst men and women born very preterm, twins had an increased likelihood of reproducing compared to singletons (women: HR = 1.25, 95% CI = 1.02-1.62; men: HR = 1.34, 95% CI = 1.01-1.78).

**Conclusions:**

Twins have lower reproduction rates compared to singletons, which only to a certain degree can be explained by diverging birth characteristics.

## Background

Worldwide there has been a steady increase of twin births in Sweden the rate rose from 8.2 births/1000 women in 1973 to 16.3 births/1000 women in 2003 [1-3]. Lately this increase can be attributed to IVF-treatment and hormone stimulation but also to the increasing maternal age [2,4]. This in combination with the improvement of medical treatment of preterm and/or small for gestational age (SGA) children has contributed to a higher number of surviving twins. In many cases twins are born preterm and with a low birthweight [5].

Ekholm et al. [6] showed that women born with very low birthweight had a reduced likelihood of reproducing. On the other hand, women born SGA had an increased likelihood of reproducing. A later study on partially the same but now enlarged material confirmed the decreased likelihood of reproducing for both men and women born preterm, but not the increased likelihood for women born SGA who seemed to start having children at an earlier age than the average population [7]. Slightly contradictory findings were presented by Swamy et al. [8] who found a decreased likelihood for reproducing amongst both women and men born preterm or SGA.

Other researchers have found that growth restriction and preterm birth can alter reproductive organs and functioning and hence, in theory, can affect future fertility and therefore have an impact on reproduction [9,10]. Also, females born SGA have been found to have reduced ovarian and uterus size as well as reduced ovulation rate [10]. It has also been indicated that males born with low birthweight may have a reduced fertility [9].

Very few studies have focused on the potential effect twinning may have on future reproduction. However, studies have shown that twins marry/form relationships later in life in comparison to singletons, which could have an impact on their reproduction [11]. In addition, Lumma et al. [12] showed that females born in an opposite sex twin pair were less likely to reproduce compared to females’ part of a same sex twin pair.

It is of importance to verify if the reproductive pattern in twins truly differs from the reproductive pattern amongst singletons. If so, it is of interest to establish whether this may be due to the prematurity/low birth weight of twins or the twinning itself. Using national registers, we were able to identify 16561 persons as being twins – which approximately equals 8000 twin pairs. These data provide a unique source for exploring the importance of being born a twin, compared to singletons, and its effect on the reproduction.

The primary purpose of this study was to examine whether twins exhibited a different outcome in terms of future reproduction and to what extent a potential twinning effect could be explained by differences in birth-characteristics.

## Methods

### Definitions

Several different definitions of non-optimal birth-characteristics were used, each definition reflecting different aspects of non-optimal birth characteristics. *“Small for gestational age”* (SGA) was defined as a birthweight < −2 SD of the mean weight for the gestational length [13]. *“Large for gestational”* (LGA) age was similarly defined as a birthweight > +2 SD of the mean weight for the gestational length according to the Swedish standard [13]. *“Low birthweight”* was defined as birthweight below 2500 g and *“very low birthweight”* as a birthweight below 1500 g. *“Moderate preterm birth”* was defined as being born between gestational week 32 and 36, and *“very preterm birth”* as being born before gestational week 32. “*Educational level*” was separated in to three categories: ‘elementary school’, ‘high school’ and ‘graduate/postgraduate’. “*Parental country of origin”* was dichotomized into ‘both parents from Nordic countries’ and ‘one or both from non-Nordic countries’. *“Mother’s marital status”* had three levels: ‘married’, ‘unmarried’ and ‘divorced/widowed’. *“Parity”* was defined as either primiparity’ or ‘multiparity’. “*Mother’s age when giving birth*” was separated into four categories: 13–19 years, 20 – 26 years, 27 – 33 years and ≥ 34 years*.* “*Non-optimal birth characteristics*” include SGA, LGA and preterm delivery and “*optimal birth characteristics*” those not SGA, LGA or preterm. Missing values on country of origin and marital status were all imputed with the most common value for each variable.

### Data

The data for this study were retrieved from Swedish population-based registries. All Swedish residents are assigned unique personal identification numbers, which enables us to individually link the information in the different registers. All registers have been validated.

· The Swedish Medical Birth Register (MBR): Medical information on all births since 1973 and onwards has been stored in the MBR and is held by the Swedish National Board of Health and Welfare [14].

· The Total Population Register (TPR) and the Multi-Generation Register: The TPR is held by Statistics Sweden and was established in 1968 [15,16]. The register contains information on variables such as births, deaths, migrations, and marital status. The Multi-Generation Register is founded on information from the TPR and enables us to identify the fathers of the studied women (in the MBR only information on the mother is included).

· The Causes of Death Register: The Causes of Death Register, which is held by the Swedish National Board of Health and Welfare, contains information on the cause of death and was established in 1961 [17].

· The Education Register and the Population and Housing Census: Since 1985, Statistics Sweden has continuously collected information on the educational level of the population in the Education Register [18,19].

Our study population was ‘twins and singletons born and still living in Sweden at the age of 13’. However, MBR registers all births in Sweden and hence an extensive and careful cleaning of the data was required to remove individuals who did not reside in Sweden at the age of 13 as well as to remove individuals with missing or invalid data. A total of 1 070 380 birth were registered in 1973 – 1983 according to both MBR and TPR, of these 10 811 individuals were deceased before age 13 (of which 10 023 were singletons, 776 were twins and 12 with unknown twin status). Persons with missing values on birthweight (n = 2114) and on gestational length (n = 4840) were excluded. Individuals (n = 663) that were considered to have an extremely high birthweight with respect to gestational length were excluded( > = 2000 grams for gestational weeks < =28, > = 2500 grams for gestational weeks 29 and 30, > = 3000 grams for gestational weeks 31 and 32, > = 3500 grams for gestational weeks 33 and 34. Birthweights for other gestational weeks were within limit.). Also, 3629 cases with an unlikely low birthweight with respect to gestational length were excluded (<= 400 grams in gestational week 29, <= 800 grams in gestational week 30, <=1000 grams in gestational week 31, <= 1150 grams in gestational week 32, <= 1250 grams in gestational week 33, <= 1450 grams in gestational week 34, <= 1600 grams in gestational week 35, <= 1700 grams in gestational week 36, <= 1800 grams in gestational week 37, <= 1950 grams in gestational week 39, <= 2000 grams in gestational weeks 40, 41 and 42, <= 2500 grams in gestational weeks 43 and 44). Furthermore, we had to exclude persons who had emigrated, and not returned to Sweden, before the age of 13 (n = 20 507) or immigrated to Sweden at age 14 and onwards (n = 5485). Finally, 5423 individuals were removed due to lack of information on the father of the child. The final data set, after removal of triplets (n = 220), comprised of 1 016 688 individuals born as singleton or twin, out of these individuals a total of 16 561 twins could be identified. These individuals were followed until the end of 2006.

### Statistical analysis

In order to investigate whether being born as a twin was related to later childbearing (measured as the hazard ratio for giving birth) we analysed the data through Cox’s proportional hazards model. Individuals exited from the models when they gave birth to the first child, emigrated, died or reached the end of the follow-up. Adjustments were made for socio demographic characteristics such as parental educational level, age at giving birth, marital status and parity, and in a separate analysis adjustments were also made for their own birth characteristics. To account for the possible age effect on the outcome analyses were also performed on data stratified into three groups based on year of birth (1973–1975, 1976–1979 and 1980–1983 which equal ages 31–33, 27–30, and 23–26 at the end of the study period). The analyses were done by gender. All analyses were performed using SPSS, version 19.0 (IBM SPSS Inc., Armonk, NY).

### Details of ethic approval

Human Research Ethics Committee. Faculty of Health Sciences, Linköping University no. 03–556, 03–557, 07-M66 08 – 08-M 233–8.

## Results

### Socio-demographic and birth characteristics in twins and singletons

Significant differences were found, for both males and females, on all socio-demographic variables, except parental country of origin, when comparing twins and singletons. Female and male twins had parents with a higher level of education, their mothers were more often married, somewhat older and had more often had previous children compared to singletons (Table 1). As could be expected considerable differences were found for both females and males on *“size for gestational age”*, *“birthweight”* and *“gestational week”* between twins and singletons (Table 1). Male and female twins were more often born SGA, they were more often born with a low or very low birthweight and also more often born premature compared to singletons.

**Table 1:** Parental socio-demographic characteristics, year of birth and birth characteristics of the studied twins and singletons, by gender

	**Females**					**Males**				
	**Singletons**		**Twins**			**Singletons**		**Twins**		
	**n**	**%**	**n**	**%**	**p-value**	**n**	**%**	**n**	**%**	**p-value**
**Mother’s educational level**					<0.001					<0.001
Missing*	32 269	6.6	533	6.5		33 850	6.6	567	6.8	
9-10 years	139 153	28.6	2371	28.7		146 638	28.5	2331	28.0	
11-13 years	205 252	42.2	3388	41.1		216 573	42.2	3338	40.2	
≥14 years	109 652	22.6	1956	23.7		116 740	22.7	2077	25.0	
**Father’s educational level**					<0.001					<0.001
Missing*	36 483	7.5	534	6.5		38 355	7.5	605	7.3	
9-10 years	153 488	31.6	2625	31.8		161 647	31.5	2567	30.9	
11-13 years	193 682	39.8	3247	39.4		204 833	39.9	3238	38.9	
≥14 years	102 673	21.1	1842	22.3		108 966	21.2	1903	22.9	
**Mother’s civil status**					<0.001					<0.001
Divorced/widowed	16 938	3.5	349	4.2		17 752	3.5	378	4.5	
Unmarried	132 503	27.2	1960	23.8		139 369	27.1	1953	23.5	
Married	336 885	69.3	5939	72.0		356 680	69.4	5982	72.0	
**Parental country of origin**					0.971					0.930
One or both non-Nordic	37 553	7.7	636	7.7		39 300	7.7	638	7.7	
Both Nordic	448 773	92.3	7612	92.3		474 501	92.3	7675	92.3	
**Mother’s age at birth**					<0.001					<0.001
13-19 years	27 291	5.6	284	3.4		28 543	5.6	285	3.4	
20-26 years	215 608	44.3	3137	38.0		227 765	44.3	3164	38.1	
27-33 years	194 398	40.0	3726	45.2		205 474	40.0	3748	45.1	
≥34 years	49 029	10.1	1101	13.3		52 019	10.1	1116	13.4	
**Mother’s parity**					<0.001					<0.001
Previous children	278 112	57.2	5434	65.9		294 131	57.2	5659	68.1	
No previous children	208 214	42.8	2814	34.1		219 670	42.8	2654	31.9	
**Year of birth**					<0.001					<0.001
Mean/SD	1977.82/3.18		1978.00/3.16			1977.82/3.18		1978.05/3.19		
**Size for gestational age**					<0.001					<0.001
Appropriate for gestational age	455 865	93.7	6228	75.5		481 038	93.6	6652	80.0	
Small for gestational age	18 573	3.8	1985	24.1		18 213	3.5	1614	19.4	
Large for gestational age	11 888	2.4	35	0.4		14 550	2.8	47	0.6	
**Birthweight**					<0.001					<0.001
Normal birthweight (> = 2500 g)	470 607	96.8	4860	58.9		499971	97.3	5411	65.1	
Low birthweight (1500–2499 g)	14 661	3.0	3186	38.6		12840	2.5	2725	32.8	
Very low birthweight (<1500 g)	1058	0.2	202	2.4		990	0.2	177	2.1	
**Gestational age**					<0.001					<0.001
Term (37–42 weeks)	455 742	93.7	5406	65.5		478 279	93.1	5400	65.0	
Preterm (32–36 weeks)	17 399	3.6	2548	30.9		21 909	4.3	2578	31.0	
Very preterm (<32 weeks)	1539	0.3	251	3.0		1779	0.3	294	3.5	
Post term (>42 weeks)	11 646	2.4	43	0.5		11 834	2.3	41	0.5	

*Parents with missing information on education were mainly immigrants and therefore included as a separate category in the analysis.

### Univariate analysis of reproduction in twins and singletons

Unadjusted hazard ratios for twins in comparison to singletons were 0.87 (95% CI = 0.84-0.90) for females and 0.89 (95% CI = 0.85-0.94) for males. Estimated reproductive rates till the age of 30 were amongst female twins and singletons 40% and 44%, respectively. Corresponding rates for male twins and singletons were 24% and 26%.

### Multivariate analysis of reproduction in female twins vs. female singletons

Increasing maternal age and no previous children were associated with a lower likelihood of reproducing amongst female twins as well as singletons (Table 2). Adjusting for socio-demographic factors, female twins had a decreased likelihood of becoming a parent compared to female singletons (HR = 0.89 95% CI = 0.86-0.93, Table 2). Adjusting also for birth characteristics resulted in minor changes in the relationship between socio-demographic background and the likelihood of reproducing (HR = 0.97 95% CI = 0.88-0.95). When stratifying by age when giving birth it was found that female twins had a reduced likelihood of reproducing compared to female singletons both before and after the age of 25 years. Also when stratifying on year of birth, the likelihood of reproducing amongst female twins was reduced compared to female singletons for those born 1976–1979, and 1980–1983, respectively (Table 3). Furthermore, when the analysis was stratified by gestational age, female twins had a reduced likelihood of becoming a parent compared to female singletons both amongst those born term and moderately preterm, whilst those twins born very preterm had an increased likelihood of reproducing compared to very preterm singletons (HR = 1.29, 95% CI = 1.02-1.63). A similar pattern was detected when stratifying by birth weight. Female twins who were born with a normal or (moderately) low birth weight had a decreased likelihood of reproducing compared to female singletons whilst female twins with a very low birth weight tended to have an increased likelihood of reproducing. Interaction terms between twinning and socio-demographic variables as well as twinning and birth characteristics have been tested. It was found that amongst females there were significant interactions between year of birth and twin birth (p = 0.007) and very preterm birth by twin birth (p = 0.002).

**Table 2:** Adjusted hazard ratios of reproducing (HR) in relation to twinning, parental socio-demographic characteristics and birth characteristics by gender

	**Females**				**Males**			
	**HR**^**a**^	**95% CI**	**HR**^**b**^	**95% CI**	**HR**^**a**^	**95% CI**	**HR**^**b**^	**95% CI**
**Twinbirth**	0.89	0.86-0.93	0.91	0.88-0.95	0.92	(0.87-0.97)	0.96	0.91-1.01
**Mother’s educational level**								
9-10 years	*Reference level*				*Reference level*			
11-13 years	0.84	0.83-0.85	0.95	0.94-0.97	0.89	0.88-0-91	0.84	0.83-0.85
≥14 years	0.67	0.66-0.68	0.80	0.79-0.82	0.74	0.73-0.76	0.66	0.66-0.68
Missing*	1.05	1.03-1.07	0.64	0.62-0.65	1.02	1.00-1.05	1.05	1.03-1.07
**Father’s educational level**								
9-10 years	*Reference level*				*Reference level*			
11-13 years	0.89	0.88-0.90	0.89	0.88-0.90	1.00	0.98-1.03	0.91	0.89-0.92
≥14 years	0.88	0.68-0.69	0.68	0.67-0.69	0.91	0.88-0.93	0.72	0.71-0.74
Missing*	1.01	0.99-1.02	1.01	0.99-1.02	0.72	0.70-0.74	1.00	0.98-1.02
**Mother’s civil status**								
Divorced/widowed	*Reference level*				*Reference level*			
Married	0.81	0.79-0.83	0.81	0.79-0.83	0.88	0.85-0.91	0.88	0.85-0.91
Unmarried	0.84	0.82-0.86	0.84	0.82-0.86	0.87	0.84-0.91	0.87	0.84-0.90
**Parental country of origin**								
One or both non-Nordic	*Reference level*				*Reference level*			
Both Nordic	1.34	1.32-1.37	1.34	1.32-1.37	1.20	1.17-1.23	1.20	1.17-1.23
**Mother’s age at birth**								
≥34 years	*Reference level*				*Reference level*			
13-19 years	2.25	2.19-2.31	2.25	2.19-2.30	2.08	2.02-2.20	2.08	2.02-2.16
20-26 years	1.59	1.56-1.62	1.58	1.55-1.61	1.56	1.52-1.60	1.55	1.52-1.59
27-33 years	1.14	1.12-1.16	1.14	1.12-1.16	1.17	1.14-1.20	1.17	1.14-1.20
**Mother’s parity**								
Previous children	*Reference level*				*Reference level*			
No previous children	0.82	0.81-0.83	0.82	0.81-0.83	0.83	0.82-0.84	0.83	0.82-0.84
**Year of birth**^**$**^	1.03	1.03-1.03	1.03	1.03-1.03	0.94	0.94-0.94	0.94	0.94-0.94
**Size for gestational age**								
Appropriate for gestational age	*Reference level*				*Reference level*			
Small for gestational age			1.02	1.00-1.05			0.97	0.94-1.01
Large for gestational age			1.02	0.99-1.05			1.03	0.99-1.07
**Birthweight**								
Normal birthweight (> = 2500 g)	*Reference level*				*Reference level*			
Low birthweight (1500–2499 g)			0.97	0.94-1.00			0.95	0.91-1.00
Very low birthweight (<1500 g)			0.87	0.75-1.00			1.03	0.85-1.24
**Gestational age**								
Term (37–42 weeks)	*Reference level*				*Reference level*			
Preterm (32–36 weeks)			0.96	0.94-0.99			0.97	0.94-1.00
Very preterm (<32 weeks)			0.88	0.78-0.99			0.78	0.68-0.90
Post term (>42 weeks)			1.01	0.98-1.04			0.93	0.97-1.04

*Parents with missing information on education were mainly immigrants and therefore included as a separate category in the analysis.

^$^Included in the model as a continuous variable.

^a^Adjusted for socio demographic variables presented in Table 1.

^b^Adjusted for socio demographic and birth characteristics presented in Table 1.

**Table 3:** Adjusted hazard ratios of reproducing in twins vs. singletons (HR) in males and females and in sub-groups defined by age and birth characteristics

	**Female**				**Male**			
	**HR**^**a**^	**95% CI**	**HR**^**b**^	**95% CI**	**HR**^**a**^	**95% CI**	**HR**^**b**^	**95% CI**
**Age**								
<= 25 years	0.86	(0.81-0.91)	0.92	(0.86-0.97)	0.91	(0.84-0.98)	0.90	(0.84-0.98)
>25 years	0.91	(0.87-0.96)	0.95	(0.90-1.00)	0.93	(0.87-0.99)	1.01	(0.94-1.08)
**Year of birth**								
1973-1975	0.93	(0.88-0.98)	0.96	(0.91-1.02)	0.96	(0.89-1.03)	1.01	(0.94-1.09)
1976-1979	0.87	(0.82-0.92)	0.88	(0.83-0.94)	0.89	(0.82-0.96)	0.92	(0.84-1.00)
1980-1983	0.83	(0.75-0.91)	0.82	(0.74-0.91)	0.84	(0.72-0.99)	0.84	(0.72-0.98)
**Gestational age**								
Term (37–42 weeks)	0.92	(0.88-0.96)	0.92	(0.88-0.96)	0.94	(0.89-1.00)	0.97	(0.91-1.03)
Post term (>42 weeks)	0.72	(0.46-1.15)	0.70	(0.44-1.12)	0.98	(0.55-1.79)	1.03	(0.57-1.88)
Preterm (32–36 weeks)	0.85	(0.78-0.91)	0.86	(0.79-0.92)	0.89	(0.80-0.98)	0.90	(0.81-0.99)
Very preterm (<32 weeks)	1.28	(1.02-1.62)	1.29	(1.02-1.63)	1.34	(1.01-1.78)	1.33	(1.00 -1.77)
**Birthweight**								
Normal birthweight (≥2500 g)	0.90	(0.85-0.94)	0.90	(0.86-0.94)	0.92	(0.86-0.98)	0.92	(0.87-0.98)
Low birthweight (1500–2499 g)	0.91	(0.86-0.97)	0.92	(0.86-0.98)	1.05	(0.96-1.16)	1.04	(0.95-1.15)
Very low birthweight (<1500 g)	1.11	(0.84-1.46)	1.10	(0.83-1.46)	0.81	(0.52-1.25)	0.79	(0.51-1.24)
**Size for gestational age**								
Appropriate for gestational age	0.87	(0.84-0.91)	0.89	(0.85-0.93)	0.91	(0.86-0.96)	0.93	(0.88-0.99)
Small for gestational age	0.93	(0.86-1.00)	0.96	(0.88-1.03)	1.04	(0.92-1.16)	1.09	(0.97-1.23)
Large for gestational age	1.15	(0.70-1.88)	1.22	(0.74-2.02)	0.72	(0.34-1.50)	0.78	(0.36-1.69)
**Optimality at birth**								
Optimal^$^	0.90	(0.86-0.93)	0.90	(0.86-0.93)	0.92	(0.87-0.97)	0.92	(0.87-0.97)
Non-optimal^$$^	0.88	(0.86-0.95)	0.91	(0.86-0.96)	0.95	(0.88-1.02)	0.99	(0.92-1.07)

^a^Adjusted for socio demographic variables presented in Table 1.

^b^Adjustedfor socio demographic and birth characteristics presented in Table 1.

^$^Optimal birth characteristics include persons who were not SGA, LGA or preterm at birth.

^$$^Non-optimal birth characteristics include those who were born SGA, LGA and preterm.

### Multivariate analysis of reproduction in male twins vs. male singletons

As for females, increasing maternal age and low maternal parity were associated with a lower likelihood of reproducing amongst male twins and singletons (Table 2). Male twins had a reduced likelihood of reproducing compared to male singletons (HR = 0.92 95% CI = 0.87-0.97, Table 2). However, also adjusting for birth characteristics this difference was reduced and was no longer statistically significant (HR = 0.96 95% CI = 0.91-1.01). Furthermore, males hazard ration was not statistically significant different from the hazard ration for females (p = 0.100). Stratifying on age when becoming parent, male twins had a reduced likelihood of becoming a parent in the lower age group compared to male singletons. Similar results appeared when data was stratified by year of birth though the reduction in the two oldest birth cohorts was not statistically significant (Table 3). When the analysis was stratified by gestational age, the results were very similar to that for females’ i.e. male twins born very preterm had an increased likelihood of reproducing compared to very preterm singletons whilst amongst those born term or moderately preterm male twins had a lower birth rate compared to male singletons. Male twins with normal birth weight showed reduced reproduction compared to singletons whilst male twins with moderately low weight did not show any reduction compared to low birth weight male singletons. Also in males, interaction terms between twinning and socio-demographic variables as well as twinning and birth characteristics have been tested. It was found that amongst males there was a significant interaction between year of birth by twin birth (p = 0.040) and very preterm birth by twin birth (p = 0.026).

## Discussion

Twins are very often excluded from epidemiological studies due to a suspicion that they behave differently compared to singletons [8,20-22]. In this study the main focus was to compare twins’ and singletons’ reproduction rates in order to explore potential differences especially in relation to birth characteristics.

We found that twins have a lower reproductive rate compared to singletons. One possible explanation is that other birth characteristics such as prematurity and size for gestational age are of more importance when analysing reproduction rates. Socio-demographic and birth characteristics do have an effect on reproduction rates. Nevertheless, a difference still remains between twins and singletons, which has not been accounted for. It could be that twins rely on one another for companionship and hence, as studies have reported, marry/form relationships later in life compared to singletons, which could affect their reproductive pattern [11,23]. Also, although the models are adjusted for socio-demographic factors such as, parental education, marital status, age when giving birth and birth characteristics, additional confounding socio-demographic factors not accounted for in the models may be of importance [24]. In a Swedish study, Hjern et al. [25] found that twins more often had a university degree than singletons after adjustment for parental socio-economic status .Whether this could explain, or is a consequence of, the lower reproduction rate in twins is unclear and assessing this would require a separate study.

Furthermore, male and female twins, born at term or moderately preterm, were found to have a reduced likelihood of reproducing compared to singletons of the same category. However, twins, male and female, born very preterm had an increased likelihood compared with singletons born very preterm. The reason for these findings can be related to differences in aetiology for preterm birth and thus the risk for complications, later related to reproductive capacity. Singletons are mostly born preterm due to pregnancy complications e.g. infections, preeclampsia etc. whilst twins more often are delivered preterm due to mechanical reasons [26].

In the analysis stratified by year of birth we found that twins had lower reproductive rates than singletons in all cohorts. The lower rate in twins appear to persist amongst females also after the age of 25 but it should be remembered that in this study the individuals are aged between 23 to 33 at end of follow-up. We are therefore only following the women through about half of their reproductive years and less so for males since they, on average, become parents at an older age than females.

In 2011 deKeyser et al. [7] reported different likelihoods of reproducing amongst males and females if they were born extremely preterm, SGA and with low weight, justifying gender specific analysis. Moreover, we found few interactions between twinning and other variables (significant interactions were found between twinning and year of birth as well as between twinning and gestational week). There seems to be a slight reduction in the difference in birth-rates between twins and singletons over age though not always achieving statistical significance (p = 0.04 amongst males and p = 0.40 amongst females).

This is a population based study in which all children born in 1973–1983 who were still alive and living in Sweden at 13 years of age were included. Due to the aim of the study data related to the study populations’ own births as well as the birth of their firstborn were collected from The Swedish National Board of Health and Welfare. The major strength of this study is that it is a population based and therefore all analyses are based on a large number of individuals. Another strength is that all data are prospectively collected from registers maintained by The Swedish National Board of Health and Welfare and Statistics Sweden. These registers are evaluated regularly and in the last evaluation, performed in 2003, it was confirmed that MBR covers 99% of all newborns, birth weight is missing for only 0.04-1.95%, gestational length has a 97% correspondence. However, there is a possibility that gestational length have been miscalculated in both ways, i.e. both overestimating as well as underestimating the gestational age at time of delivery. This is most markedly evident in the post-term group. The reporting to NPR is complete for inpatient hospital care and 98.6% of all incidents are correctly classified. Also, through the unique personal identification number assigned to each individual we were able to link MBR-data to socio-demographic variables, which allowed for thorough/extensive analysis.

This data did not allow us to control for monozygotic and dizygotic twins since the registers did not contain this information. Hence, in the comparison of male singleton with male twin, the male twins’ co-twin is in most cases a female (and the reverse relationship when comparing female singleton to female twin). On the other hand the primary goal in this study was to analyse potential differences between twins and singletons, regardless of the twin being homozygote or heterozygote. Also, though not part of the aim of the study, another limitation of this study is that no analysis has been performed comparing the reproductive rates between twins raised as twins and twins raised as singletons. Finally, since the study population was defined as persons born between 1973 and 1983 the twinning rate in this study was not affected by assisted reproductive treatments.

## Conclusions

Twins, both males and females, were found to have a lower reproduction rate compared to singletons, when adjusting for socio-demographic factors. This difference in birth rates can partly be explained by diverging birth characteristics.

For future research it will be of great interest to follow the growing population of twins born after IVF-treatment and their reproductive patterns.

## Competing interests

The authors declared that they have no competing interests.

## Authors’ contribution

GS and OF had the original idea for the study. All authors planned the study. MB analysed the data and drafted the paper. All authors contributed to the interpretation of the data, revisions and gave input at all stages of the study. All authors have approved to the final version of the manuscript.

## Pre-publication history

The pre-publication history for this paper can be accessed here:

http://www.biomedcentral.com/1471-2393/13/6/prepub
